# *Aspergillus*-*Penicillium* co-culture: An investigation of bioagents for controlling *Fusarium proliferatum*-induced basal rot in onion

**DOI:** 10.3934/microbiol.2024044

**Published:** 2024-11-19

**Authors:** Mohammed M. M. Abdelrahem, Mohamed E. Abouelela, Nageh F. Abo-Dahab, Abdallah M. A. Hassane

**Affiliations:** 1 Department of Botany and Microbiology, Faculty of Science, Al-Azhar University, Assiut Branch, Assiut 71524, Egypt; 2 Department of Pharmacognosy, Faculty of Pharmacy (Boys), Al-Azhar University, Cairo 11884, Egypt

**Keywords:** co-culture, *Aspergillus*, *Penicillium*, *Fusarium* basal rot, onion, HPLC profiling, principal component analysis

## Abstract

Fungal co-culture is a method that allows the detection of interactions between fungi, enabling the examination of bioactive novel metabolites induction that may not be produced in monocultures. Worldwide, *Fusarium* basal rot is a primary limitation to onion yield, being caused by different *Fusarium* species. Current research directions encourage biological control of plant diseases as a replacement for routine chemical treatments. The current study aimed to investigate the co-culturing technique for mining new sources of bioagents that could be used as fungicides. *Aspergillus ochraceus* AUMC15539 was co-cultured with *Penicillium chrysogenum* AUMC15504, and their ethyl acetate extract was tested in vitro and in a greenhouse against *Fusarium proliferatum* AUMC15541. The results showed that *Aspergillus*-*Penicillium* (AP) co-culture extract significantly inhibited the growth of *F. proliferatum* with an MIC value of 0.78 mg/mL and showed antioxidant efficiency with an IC_50_ value of 1.31 mg/mL. The brine shrimp toxicity testing showed a LC_50_ value of 2.77 mg/mL. In addition, the co-culture extract showed the highest phenolic content at 114.71 GAE mg/g, with a 27.82 QE mg/g flavonoid content. Profiling of AP co-culture and its monoculture extracts by HPLC revealed a change in the metabolites profile in AP co-culture. Principal component analysis verified a positive correlation between the obtained HPLC data of *A. ochraceus* (A), *P. chrysogenum* (P), and AP extracts. Greenhouse experiments demonstrated that treating infected onion plants with the AP co-culture extract significantly enhanced all growth parameters. Additionally, the co-culture extract treatment resulted in the highest levels of total pigments (3.46 mg/g), carbohydrates (52.10 mg/g dry weight), proteins (131.44 mg/g), phenolics (41.66 GAE mg/g), and flavonoids (9.43 QE mg/g) compared with other treatments. This indicates a promising potential for fungal co-cultures in discovering new bioagents with antifungal properties and growth-promoting capabilities.

## 1. Introduction

Microorganisms play a crucial role as sustainable and promising candidates for producing novel marketable bioactive materials with a wide range of biodiversity and chemodiversity [1]–[3]. Many microbes secrete antibiotics or antifungal substances to defend themselves against nearby harmful microorganisms and to compete for space and resources [4]. Among microorganisms, fungi contribute approximately 45% of all valuable bioactive microbial metabolites [3]. Endophytic fungi are a polyphyletic group that asymptomatically inhabit plant tissues, exhibiting remarkable diversity and representing a rich source of bioactive natural products [5]. The major classes of bioactive compounds produced by endophytic fungi include alkaloids, steroids, peptides, phenols, terpenoids, flavonoids, and quinines [6]. These metabolites protect their hosts from pathogens and harsh environments, while also producing compounds with therapeutic potential [7],[8]. Fungal species isolated from different plant rhizospheres are of special interest given their ability to generate interactions with plant roots to ensure the absorption of nutrients and protection against other microbial pathogens, with an impact on plant growth and nutrition [9],[10].

Endophytes and epiphytes isolated from plants naturally compete with plant pathogenic fungi [11]. This competition for resources is thought to activate silent biogenetic gene clusters, leading to the production of cryptic secondary metabolites that are undetectable in axenic cultures [12]. The one strain–many compounds (OSMAC) strategy is a powerful technique in natural product discovery, enabling researchers to exploit the full metabolic capabilities of microorganisms by adjusting cultivation conditions, utilizing co-cultivation, and applying chemical elicitors. Additionally, the stimulation of silent genes enhances the chemodiversity of the microorganisms at the genome, transcriptome, proteome, or metabolome levels [13]. This approach not only broadens the spectrum of metabolites produced but also enhances the potential for discovering new drugs and bioproducts [14].

The co-culture technique, which involves growing two or more microorganisms together, is an OSMAC strategy method that induces biotic stress through interactions with other microbial partners, resulting in the production of compounds for defense or nutrient competition mechanisms [15]. In a co-culture experiment, microbial communication occurs either via volatile compounds or in loco signaling and leads to the regulation of specialized metabolites. This technique has been shown to induce chemodiversity without requiring any prior knowledge of the genome or any special equipment for cultivation and data interpretation [16]. Microbial interactions in co-cultures can be classified into positive, neutral, or negative effects, depending on how one microbe influences another [17]. These effects vary according to the challenge strain, providing different chemical and biological outcomes. When it comes to bioactivity, many studies focus on increasing the amount and variety of antimicrobial substances through competitive interactions in co-culture systems [18].

*Aspergillus* is a well-known genus for its chemodiversity. The co-culture of *Aspergillus* species with various bacteria and fungi has led to the discovery of several newly described and up-regulated compounds, being the most studied inducer fungus reported in mixed fermentations. *Penicillium* is a diverse genus occurring worldwide and with large quantities of diverse secondary metabolites exhibiting potent bioactivities [19]. In most co-culture experiments, metabolite extraction is performed using conventional protocols that use organic solvents [11]. The conventional method for the study of metabolites in co-culture systems is to separate and purify the compounds corresponding to each peak newly detected in GC and HPLC and analyze their structures by means of MS, UV, IR, and NMR [20].

Onion (*Allium cepa* L.), belonging to the Amaryllidaceae family, is one of the most important commercial vegetable crops and is widely grown in almost all countries around the world [21]. Onion is known for its medicinal properties due to the presence of flavonoids, anthocyanins, fructooligosaccharides, and organosulfur compounds [22]. Onions also provide carbohydrates, proteins, vitamins, and minerals [23]. They are used in various forms, including as spice, pickle, juice, or raw in salads [24].

Among the fungal pathogens that affect the *Allium* species, *Fusarium* basal rot (FBR) is a major limitation to *Allium* production worldwide. The disease has been associated with different species of *Fusarium*, including *F. oxysporum*, *F. proliferatum*, *F. solani*, *F. acuminatum*, *F. redolens*, *F. verticillioides*, *F. equiseti*, *F. culmorum*, *F. falciforme*, and *F. brachygibbosum*, from which *F. oxysporum* and *F. proliferatum* are the most prevalent. Various agronomic practices have been used to control *Fusarium* basal rot, including the use of resistant onion cultivars, long crop rotation, solarization, treatments of seed with fungicides, and soil fumigation [25]. Although the use of pathogen-resistant cultivars may reduce the damage caused by *Fusarium*, variations in the aggressiveness and genetic structure of the pathogen make it difficult to breed resistant cultivars [26]. Synthetic pesticides have been a major crop protection tool since the 1960s, but they have negative effects on people, animals, and the environment, which led to their banning in many countries [27],[28]. Alternatively, natural products have been studied to control fungal diseases and reduce the use of synthetic fungicides [29]. The development of phytopathogen resistance, as well as legal restrictions to conventional pesticides, underscore the need for alternative natural, safe, and effective plant protection agents [30].

The current study explores the use of fungal co-cultures, specifically *Aspergillus ochraceus* and *Penicillium chrysogenum*, as new sources of bioagents with potential fungicidal properties against *Fusarium* basal rot infection in onion caused by *Fusarium proliferatum* either *in vivo* and in a greenhouse. Analysis of metabolite profiles by HPLC profiling of the mono- and co-culture extracts was carried out along with the evaluation of their effects on vitality parameters of infected onion plants as health indicators, including total pigments, carbohydrates, proteins, phenolics, and flavonoids.

## 2. Materials and methods

### 2.1. Fungal isolates

Fungal isolates were obtained from the Mycology Laboratory culture collection at the Botany and Microbiology Department, Faculty of Science, Assiut Branch, Al-Azhar University, Egypt. *Aspergillus ochraceus* MLBM405 (AUMC 15539) [31], a fungal endophyte derived from *Allium cepa* (onion), and *Penicillium chrysogenum* MLBM705 (AUMC15504), a rhizospheric fungus associated with onion plant, were cultivated on autoclaved glucose-Czapek's agar (Cz) with a pH 6.5 (NaNO_3_, 2 g; KH_2_PO_4_, 1 g; MgSO_4_.7H_2_O, 0.5 g; KCl, 0.5 g; glucose, 10 g, agar, 20 g per liter of distilled water) and potato dextrose agar (PDA): 200 g of potatoes are boiled in 1 L of distilled water and sieved, glucose (20 g/L) and agar (20 g/L) were added with a pH value of approximately 5.6 [32], and incubated for 7–10 days at 28 °C. *Fusarium proliferatum* AUMC15541 causing *Fusarium* basal rot (FBR) in onion was used as test phytopathogen for dual culture plate assay (co-culture assessment) and cultivated on Cz and PDA medium.

### 2.2. Molecular identification of the selected fungal isolates

#### 2.2.1. Isolation of genomic DNA

Czapek-Dox broth medium was utilized to cultivate pure fungal isolates for 5 days at 28 °C. Then, total DNA was extracted from each isolate using the Norgen Plant/Fungi DNA Isolation Kit (Sigma, Thorold, Canada) following the method described by Hassan et al. [33]. After elution, the DNA was kept at −20 °C for further amplification.

#### 2.2.2. PCR amplification and nucleotide sequencing

The ITS region of selected strains was amplified using specific universal primers ITS-1 (5′-TCC GTA GGT GAA CCT GCG G-3′) and ITS-4 (5′-TCC TCC GCT TAT TGA TAT GC-3′) as suggested by Mohamed et al. [34]. The PCR reaction was conducted as per the protocol reported by Hassan et al. [33]. The PCR tubes were filled with 25 µL of the mixture, comprising 12.5 µL of PCR master mix, 1 µL of forward and reverse primers each (approximately 20 pmol), 1 µL of genomic DNA, and 9.5 µL of ddH_2_O. The amplification of DNA was carried out using a Thermocycler C1000 Touch™ (Bio-Rad, Germany). The PCR products were then resolved on agarose gel 1.5% with TBE buffer and visualized using a gel documentation system. The PCR product was purified using the gel extraction kit (Omega Biotek) following the manufacturer's instructions. All PCR products were sequenced with the gene analyzer 3121 sequencing service (Macrogen Co., Seoul, South Korea). The obtained sequences were aligned with the BLAST search tool at the National Center for Biotechnology Information (NCBI) to detect sequence similarity. The sequences were analyzed using MegAlign (DNA Star) software, version 5.05, and compared to isolates from the sequencing databases using the BLAST search algorithm of GenBank (http://www.ncbi.nlm.nih.gov/BLAST).

### 2.3. Solid-state fermentation and extraction

A large-scale solid-state fermentation was utilized to produce fungal extracts in 1 L Erlenmeyer flasks, each filled with 100 g of rice and 110 mL of distilled water and autoclaved [35]. The fungal isolates used for co-culture and fermentation were *A. ochraceus* (A), *P. chrysogenum* (P), and *Aspergillus*-*Penicillium* (AP). For AP co-culturing, equal inoculum volumes of both fungi were used, while axenic cultures were set up for each fungus. The fermentation process was carried out by incubating flasks at 28 °C [18]. After 30 days, 500 mL of ethyl acetate was added to each flask thrice for extraction overnight. The ethyl acetate extracts were filtered and dried using a vacuum rotary evaporator (BÜCHI R-114, Switzerland) at 40 °C.

### 2.4. Antifungal assay and determination of the minimum inhibitory concentration

Fungal extracts of mono- and co-cultures were tested for their activity against *F. proliferatum* using the well-diffusion method. Wells of 8 mm diameter in pre-inoculated plates were filled with 100 µL of each extract at 100 mg concentration in dimethyl sulfoxide (DMSO) and incubated for 3–7 days at 28 °C. Bellis (commercial chemical fungicide, 100 mg/mL) was used as a positive control, while DMSO was used as a negative control. All experiments were performed three times; the diameter of the inhibition zone around the well was measured in mm using a caliper [11]. The determination of the minimum inhibitory concentration (MIC) of fungal extracts was carried out using different concentrations of fungal extracts (2-fold serial dilution ranging from 100 to 0.39 mg/mL) pipetted into wells of inoculated plates. The MIC was defined as the lowest concentration of fungal extracts that inhibited mycelial growth [36].

### 2.5. Brine shrimp cytotoxicity test

To determine the cytotoxicity of fungal extracts, the brine shrimp cytotoxicity test (BSCT) was used. The extracts were dissolved in DMSO at varying concentrations (2.5–100 mg/mL), and 100 µL of each extract was added to vials containing 4.9 mL of seawater containing 10 live brine shrimp larvae. Bellis 38% WG (BASF) and DMSO were employed as positive and negative controls, respectively. After 24 h, the number of living larvae was counted in each vial. Each extract was tested thrice, and the concentration of the extract that killed 50% of the larvae (LC_50_) was calculated [37],[38].

### 2.6. Estimation of total phenolic and flavonoid contents in fungal extracts

The total phenolic content of extracts was calculated using a modified method by Kupina et al. [39]. In this method, 1 mL of each fungal extract (10 mg/mL) was mixed with 2.5 mL of Folin–Ciocalteu reagent and 1 mL of sodium carbonate. The mixture was vortexed and incubated in the dark at room temperature for 30 min, and then the absorbance was measured at 750 nm using a UV-visible spectrophotometer (Janeway 7315, UK). The content of phenolic compounds was expressed as gallic acid equivalent (GAE mg/g) using a standard curve of gallic acid (Figure S1). To measure the total flavonoid content of fungal extracts, 0.5 mL of each extract (10 mg/mL) was mixed with 1 mL of 2% (v/v) ethanolic solution of AlCl_3_.6H_2_O. The mixture was allowed to stand for 10 min, and the absorbance was measured at 430 nm [40]. The flavonoid content was expressed as quercetin equivalent (QE mg/g) using a standard curve (Figure S2).

### 2.7. Measurement of free radical scavenging activity by 1,1-diphenyl-2-picryl-hydrazyl (DPPH) assay

The antioxidant properties of the test samples were measured as scavenging activity or hydrogen donating form based on the procedure by Brand-Williams et al. [41]. While the DPPH radical is scavenged, the color changes from purple to yellow with a decreasing 517 nm absorbance. In this method, 1.8 mL of 0.1 mM DPPH (4 mg/100 mL of methanol) mixture solution was added to 0.2 mL of the tested samples in absolute methanol at various concentrations (10, 5, 2.5, 1, 0.5, 0.1, and 0.025 mg/mL) in addition to the blank. The mixture was left aside at room temperature (shaken vigorously in-between) for 30 min and absorbance was determined by spectrophotometer (Jenway 7315) at 517 nm. Butylated hydroxytoluene (BHT) was employed as a positive control, and all measurements were performed in triplicates. The following formula (Eq 1) was utilized to determine the capacity to scavenge the DPPH radical.

$$\text{DPPH\%}=\frac{A-B}{A}\times 100,$$ (1)

where A is the negative control absorbance (methanol and DPPH) and B is the sample absorbance (DPPH, methanol, and sample). The IC_50_ was obtained by interpolation from linear regression analysis [42].

### 2.8. High-performance liquid chromatography–diode-array detection analysis (HPLC–DAD)

To understand the influence of co-culturing, HPLC was used to compare the metabolic profiles of AP co-culture with A and P monoculture extracts. Water (photodiode array) was used for HPLC analysis. Separation was achieved by using an Eclipse Plus C_18_ column with 4.6 mm × 100 mm i.d. and a particle size of 5 µm. The mobile phase consisted of 0.1% formic acid in water (A) and 0.1% formic acid in acetonitrile (B) at a flow rate of 0.8 mL/min. The mobile phase was programmed consecutively in a linear gradient. The DAD was monitored at 235, 254, 280, and 340 nm with a bandwidth of 4 nm. The column temperature was maintained at 30 °C and the sample injection volume was 10 µL [43].

### 2.9. Effect of fungal co-culture extracts in controlling *Fusarium proliferatum* on onion plants in the greenhouse

Greenhouse experiments were conducted to test the pathogenicity of *F. proliferatum*. Pot trials were designed according to the protocol adopted by Riaz et al. [44] with some modifications. Clean seedling plastic bags with a diameter of 25 cm were prepared and filled with a mixture of soil and sand (1:3), sterilized using 5% formalin solution, and well-ventilated at a rate of 2 kg/bag.

#### 2.9.1. Artificial infection and treatment with fungal extracts and fungicide

The inoculum of *F. proliferatum* was prepared by growing a pure culture of isolate on barley-washed sand (1:1, w/w) and incubated at 28 ± 2 °C. After 15 days, the fungal inoculum was added to sterilized soil at the rate of 2% (w/w). Seedling plastic bags were watered and left for one week to establish the fungal inoculum [45]. Fungal co-culture extracts (1 g/L) were prepared in DMSO, and the chemical fungicide Bellis 38% WG (BASF) was used as a comparison at a recommended dose (1 g/L water). Onion transplants (Sabeeni variety) of uniform size (2 cm diameter) susceptible to basal rot disease were surface sterilized with a 1% solution of sodium hypochlorite and thoroughly washed with sterilized water. The ends of the roots were cut to facilitate the occurrence of industrial infection. The roots and stems of the onion transplant were soaked in fungal extracts and chemical fungicide (15 plants for each treatment) for 15 min before transplantation. A set of onion transplants, not treated with fungicides or fungicide extracts, was prepared as an infectious witness (infected control), while another set, without fungal infection, was prepared as a healthy witness (healthy control). The pots were divided into groups according to each treatment [45]. Each treatment was carried out three times and the pots were irrigated when it was necessary and fertilized at recommended doses.

#### 2.9.2. Determination of microbial densities

Soil dilution and plate count technique was performed to determine the total fungal and *F. proliferatum* densities in the soil of the different treatments according to the method of Johansen et al. [46], with some modifications. Soil dilutions were made by suspending 1 g of each soil sample in 10 mL of sterile distilled H_2_O. Serial dilutions of 10^−3^, 10^−4^, and 10^−5^ were prepared and used to isolate fungi. One milliliter of each concentration was added to sterile Petri dishes containing sterile Czapeks' agar medium in triplicates. The plates were incubated at 28 ± 2 °C for 4–7 days. After that, the plates were examined and fungal colonies were counted; then, microbial densities were calculated.

#### 2.9.3. Evaluation parameters

At the end of the experiment, the plants were carefully removed from the pots, and the root systems were gently washed in tap water. Some parameters were measured to estimate the response of the onion plants to different treatments. Damage reduction rate (R% of leaves and root dry weights) was calculated according to the following Eq 2:

$$\text{R\%}=\frac{\left(\text{DWA}-\text{DWP}\right)}{\text{DWA}}\times 100,$$ (2)

where DWA is the dry weight (leaves and root) of coated plants with bioagent and DWP is the dry weight (leaves and root) of inoculated plants with only the pathogen (infested control).

The effect of the antagonist alone on the plants (D%) was also studied as the development rate of the dry leaves and root weights according to the following Eq 3:

$$\text{D\%}=\frac{\left(\text{DWA}-\text{DW}\right)}{\text{DW}}\times 100,$$ (3)

where DWA is the dry weight (leaves and root) of coated plants with bioagent and DW is the dry weight (leaves and root) of the healthy plants (non-infected control) as described by Boughalleb-M'hamdi et al. [47].

Disease incidence and plant mortality were recorded after 10 weeks of transplantation [48] according to the following Eq 4:

$$\text{DI\%}=\frac{\text{Number of infected plants}}{\text{Total number of plants}}\times 100.$$ (4)

#### 2.9.4. Morphological parameters

At the end of the experiment, plants from different treatments were removed, cleaned thoroughly with running water, and blotted with tissue paper. Plant height, root length, and leaves' length were recorded. Plants were weighed to determine fresh weight and oven-dried for 72 hours to determine dry weight [49].

#### 2.9.5. Determination of photosynthetic pigments content

The pre-weighed samples of onion tubular leaves were put separately in ethanol (20 mL per gram), grained using a mortar and pestle, homogenized using a homogenizer at 1000 rpm for approximately 5 min, and filtered using a cheesecloth. The obtained extracts were centrifuged at 5000 rpm for 10 min, the supernatants were separated, and absorbances were read at 400–700 nm using an UV-VIS spectrophotometer. Maximum absorbance of chlorophyll a is at 666 nm, chlorophyll b at 653 nm, and total carotenoids at 470 nm. The amount of present pigments was calculated according to the formulas (Eqs 5–8) by Lichtenthaler and Wellburn [50].

Chlorophyll a=15.65 A666−7.340 A653, (5)

Chlorophyll b=27.05 A653−11.21 A666, (6)

Carotenoids=1000 A470−2.860 (Chl. a)−85.9 (Chl. b)/245, (7)

Total pigments=chlorophyll a+chlorophyll b+carotenoids. (8)

The calculated pigment content was then expressed as mg/g of fresh weight as follows (Eq 9):

$$\text{Chl.a}=\frac{\text{Chl.a (µg/mL)}\times \text{Extract volume (mL)}}{\text{Fresh weight of sample (g)}\times \text{1000}}.$$ (9)

#### 2.9.6. Determination of biochemical compounds

##### 2.9.6.1. Estimation of carbohydrates

The anthrone-sulfuric acid method depicted by Fales [51] was adopted for carbohydrate determination. Fresh leaves (100 mg) were mixed in 5 mL of 2.5 N-HCl, heated in a water bath for 3 h, and then neutralized by adding sodium carbonate. Glucose was used as a standard for carbohydrate estimation and then filtered using Whatman filter paper. The extracts were used for carbohydrate determination by the anthrone method. Anthrone reagent was prepared by dissolving 200 mg of anthrone in 100 mL of ice-cold 95% H_2_SO_4_, freshly prepared before use. To 1 mL of filtrate, 5 mL of the anthrone reagent was added, mixed well by vortex, and kept at 90 °C for 10 min. After that, tubes were cooled to room temperature, and the absorbance was measured at 620 nm against a blank. The amount of carbohydrates was expressed as glucose equivalent (mg/g) using a standard curve of glucose (Figure S3).

##### 2.9.6.2. Determination of protein content

The method of Lowry [52] was used to determine total protein content by centrifuging leaf extracts [100 mg of leaf samples in 10 mL of sodium phosphate buffer (pH 7.5)] for 10 min at 10,000 rpm. The supernatant (0.1 mL) was diluted up to 1 mL, followed by shaking for 10 min after the addition of 1 mL of reagent C comprising 1 mL of copper sulfate (0.5%) and 50 mL of sodium carbonate (2 g), 0.4 g of sodium hydroxide (0.1 mol /L), and 1 g of sodium potassium tartrate dissolved in 100 mL of distilled water. After that, 0.1 mL of 50% Folin–Ciocalteu reagent was added, and samples were incubated at room temperature for 30 min. The absorbance of samples was taken at 650 nm, and various concentrations of bovine serum albumin (BSA) were used as standard. The amount of total protein was expressed as BSA equivalent (mg/g) using a standard curve of BSA (Figure S4).

##### 2.9.6.3. Estimation of total phenolic content

The total phenolic content assay was assessed using the modified procedures by Kupina et al. [39]. Briefly, milled onion leaves (0.1 g) were homogenized in 10 mL of 70% acetone and then centrifuged at 5000 rpm for 10 min; 1 mL of supernatant was treated as previously described for fungal extracts.

##### 2.9.6.4. Determination of total flavonoid content

A previous method reported by Quettier-Deleu et al. [40] was conducted to measure total flavonoid content. Milled onion leaves (0.1 g) were homogenized in 10 mL of 80% ethanol and then centrifuged at 5000 rpm for 10 min; 0.5 mL of each extract was treated as previously described for fungal extracts.

### 2.10. Statistical analysis

All experiments were carried out three times. Data were presented as the mean ± standard error (SE) and established by analysis of variance (one-way ANOVA) using the SPSS software, version 16 (IBM, Armonk, NY, USA) with multiple comparison tests (Duncan) as being below the 0.05 level of significance. Principal component analysis (PCA) was used to identify properties that explain most of the variability and to select the most appropriate indicators (a minimum data set MDS) that reflect the impact of fungal mono- and co-culture extracts. Statistical analysis was carried out using the SPSS software, and the graphical presentation was obtained using Origin 2018 software (USA, Origin Lab).

## 3. Results

### 3.1. Phylogenetic analysis of the fungal cultures

The molecular identity and phylogenetic characterization of *A. ochraceus, P. chrysogenum*, and *F. proliferatum* were determined using 18S rDNA gene sequencing (Figure S5A–C).

### 3.2. Assessment of antifungal activity, MIC, and cytotoxicity

**Figure 1.**
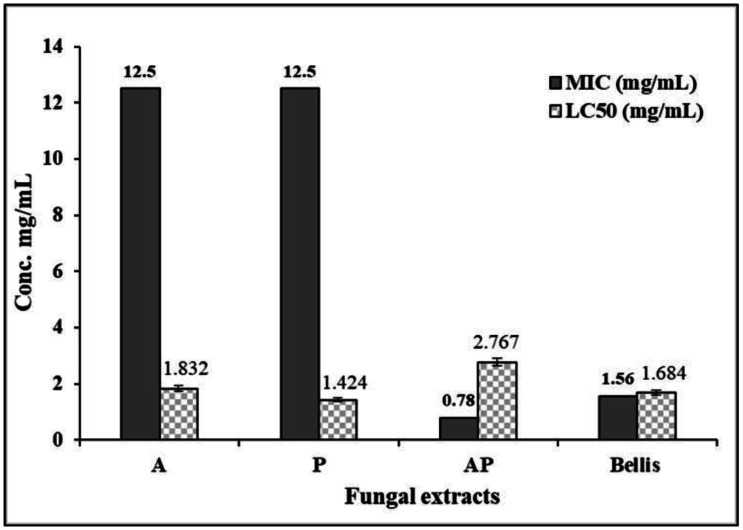
Minimum inhibitory concentration (MIC) and cytotoxic effect on brine shrimp (LC_50_) of fungal EtOAc crude extracts.

Results show that AP co-culture extract significantly inhibited the growth of *F. proliferatum*, with an inhibition zone diameter of 22.17 mm. *Penicillium chrysogenum* (P) and *A*. *ochraceus* (A) extracts presented low activity against *F. proliferatum* in comparison with their AP co-culture extract, while Bellis had the highest activity against *F. proliferatum* (Table S1). According to the obtained results, the MIC of extracts against *F. proliferatum* in AP co-culture was 0.78 mg/mL, while A and P extracts had MICs values of 12.5 mg/mL. Bellis showed a MIC of 1.56 mg/mL (Table S1, Figure 1). Regarding the LC_50_ values of brine shrimp exposed to various fungal extracts and the positive control (Bellis), the tested fungal extracts exhibited varying degrees of toxicity to brine shrimp. *Aspergillus*-*Penicillium* co-culture extract exhibited the lowest LC_50_ value (2.77 mg/mL) among all tested fungal extracts, indicating the least toxicity to brine shrimp, while A extract and Bellis extracts demonstrated moderate toxicity to brine shrimp, with LC_50_ values of 1.83 and 1.68 mg/mL, respectively. In contrast, P extract displayed high toxicity to brine shrimp, with an LC_50_ value of 1.42 mg/mL (Table S1, Figure 1).

### 3.3. Determination of total phenolics and flavonoids and antioxidant activities

**Figure 2.**
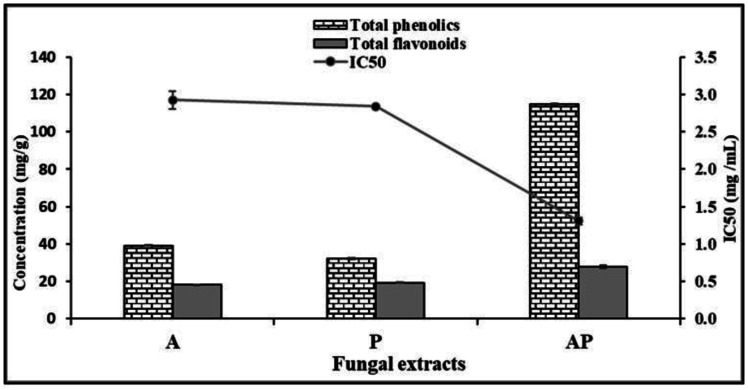
Determination of total phenolic and flavonoid contents and IC_50_ values from antioxidant of fungal extracts.

The total phenolic concentrations in GAE mg/g for different fungal extracts and their co-culture varied greatly during solid-state fermentation. The highest total phenolic content was found in AP co-culture extract (114.71 GAE mg/g). A and P extracts had total phenolics of 39.22 and 32.231 GAE mg/g, respectively. The total flavonoid content in different fungal extracts was measured in QE mg/g, with the highest being detected in AP co-culture extract at 27.82 QE mg/g. In contrast, the monoculture extracts had low total flavonoid content. The maximum scavenging radical activity IC_50_ (1.31 mg/mL) was observed in the AP co-culture. The IC_50_ value of the positive control BHT was 0.46 mg/mL using the DPPH assay (Table S2, Figure 2).

### 3.4. HPLC analysis of AP co-culture extract

Significant chemical changes were observed during the initial screening of solid-state fermentation extracts when *A. ochraceus* was co-cultured with *P. chrysogenum* and compared with the monocultures. HPLC analysis revealed alterations in the profiles of secondary products in the co-culture compared with the pure cultures. The pattern of HPLC peaks was used to identify the variations in metabolites, including new metabolite peak appearance, loss of original peak appearance, and no change in peak pattern.

**Figure 3.**
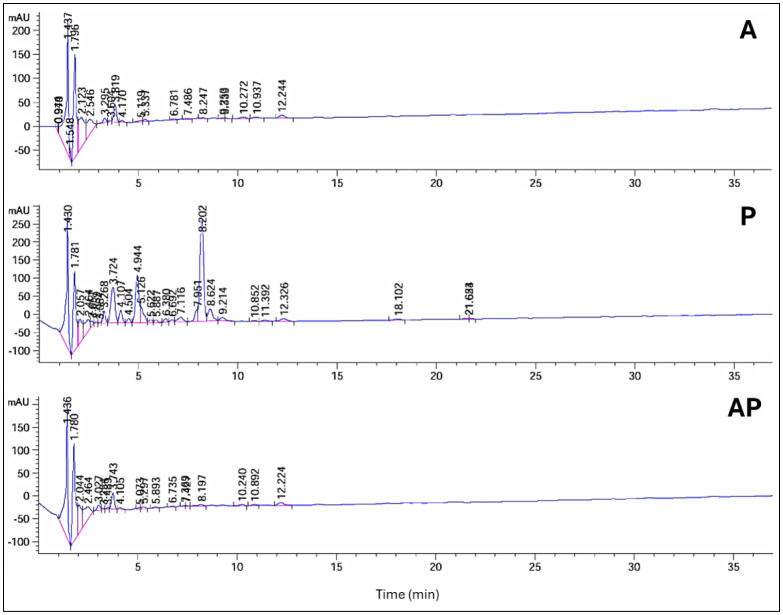
HPLC analysis of compounds produced by three different culture extracts: A, P, and AP co-culture extracts at a wavelength of 235 nm.

**Figure 4.**
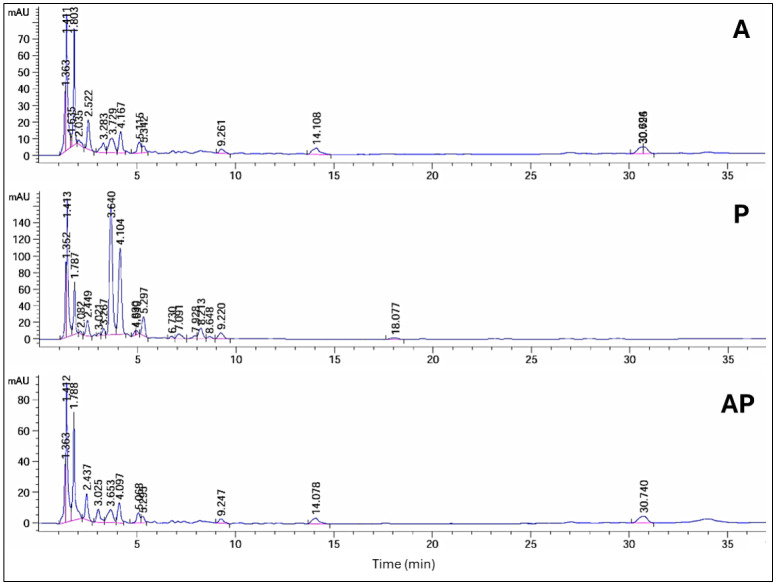
HPLC analysis of compounds produced by three different cultures: A, P, and AP co-culture extracts at a wavelength of 254 nm.

The HPLC profile at 235 nm revealed that in AP co-culture, 6 peaks' areas significantly increased, 2 peaks decreased by 3% and 75%, respectively, and 8 peak areas appeared in the A extract but not in AP. When comparing extracts from AP co-culture to axenic P culture, 1 peak area in the AP co-culture increased, 11 peaks decreased, and 13 peak areas appeared in the P extract but not in AP. Peak area at retention time (10.24 min) was detected only in AP co-culture extract (Table S3, Figure 3). The HPLC profile at 254 nm revealed that 4 peak areas of AP co-culture extract increased by 1.1, 1.2, 1.4, and 2.2-fold compared with the A extract, while 4 peak areas appeared in the A extract but not in the AP co-culture. When comparing the AP co-culture and P extracts, 3 peak areas of AP increased by 1.4, 4.3, and 2.2-fold compared with the P extract, while 7 peaks of AP co-culture decreased, and 9 peak areas appeared in the P extract but not in the AP extract (Table S4, Figure 4).

**Figure 5.**
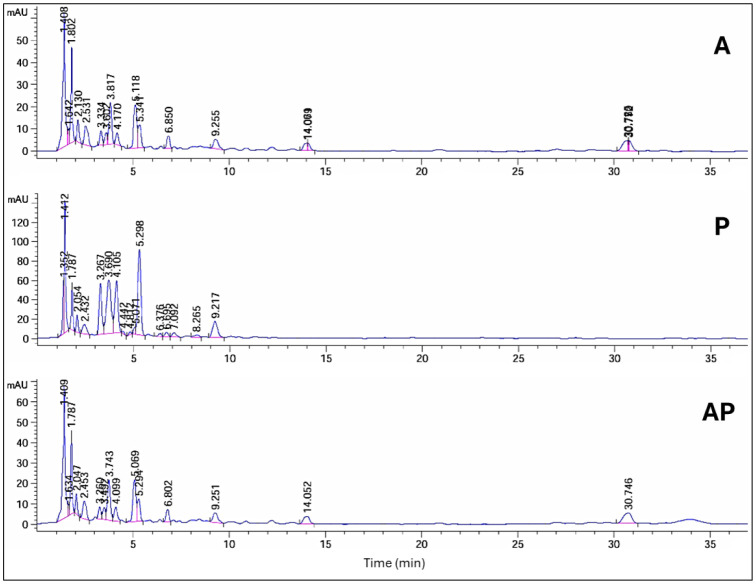
HPLC analysis of compounds produced by three different cultures: A, P, and AP co-culture extracts at a wavelength of 280 nm.

**Figure 6.**
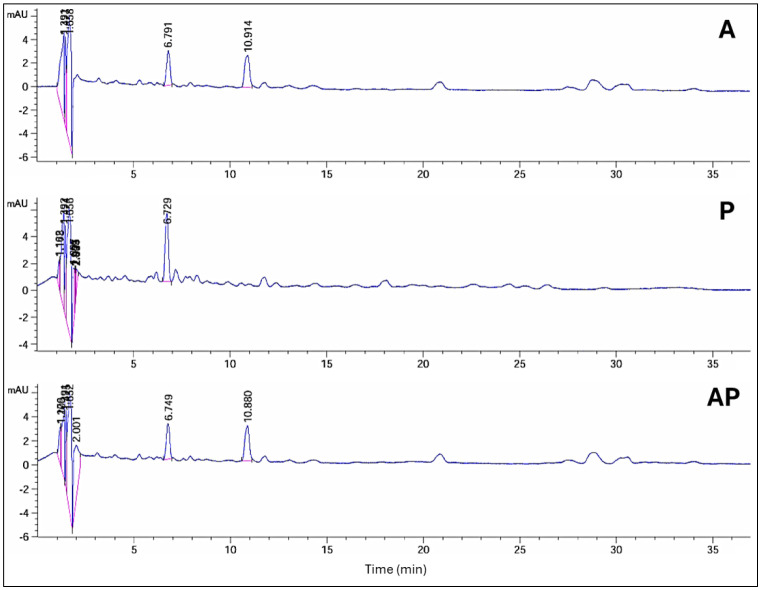
HPLC analysis of compounds produced by three different cultures: A, P, and AP co-culture extracts at a wavelength of 340 nm.

HPLC profiling at 280 nm showed that 7 peak areas of AP co-culture extract increased by 1.1, 1.2, 1.1, 1.5, 1.1, 1.7, and 1.6-fold compared with A extract, while 1 peak was visible in the A extract but not in AP. Two peak areas of AP co-culture increased by 4.2 and 1.3-fold compared with the P extract, while 9 peaks of AP extract decreased. Moreover, 6 peak areas appeared in the P extract but not in the AP extract (Table S5, Figure 5). The HPLC profile at 340 nm revealed that 4 peak areas decreased in AP, while 2 peak areas of AP co-culture increased by 1.1 and 21.2-fold compared with the P extract. Three peaks decreased by 19%, 7%, and 40% in the P extract; meanwhile, 3 peak regions were observed in the P extract but not in the AP co-culture. On the other hand, at Rt (1.17 and 1.20 min), 2 peak areas were identified in AP co-culture and not in monocultures (Table S6, Figure 6).

**Figure 7.**
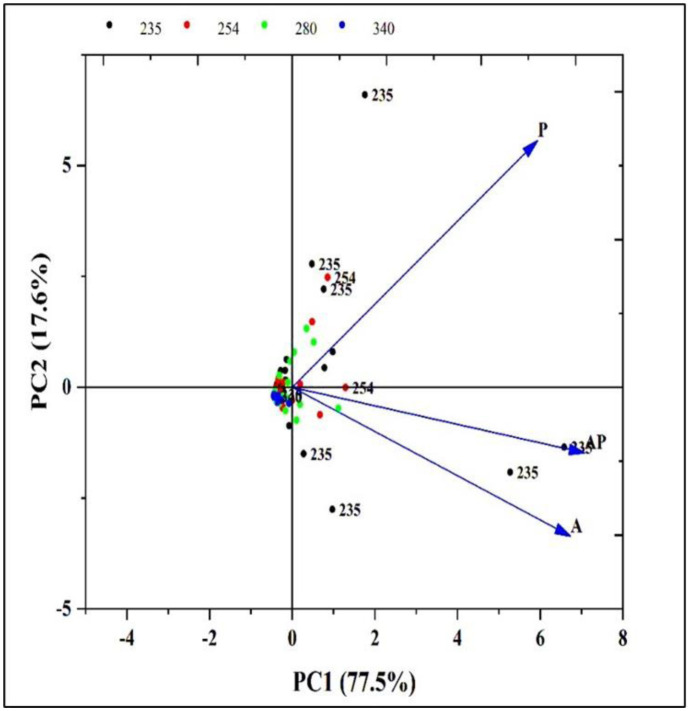
Principal component analysis (PCA) score plot of the data set obtained from HPLC analysis of A, P, and AP extracts at 235, 254, 280, and 340 nm.

A principal component analysis (PCA) was used to discover metabolic variations among A, P, and AP extracts, detected by HPLC at 235, 254, 280, and 340 nm. The PCA analysis verified a positive correlation between the obtained data of A, P, and AP extracts. The PCA graph revealed that the samples were distributed in two quadrants, which indicates chemical differences and similarities between the three extracts. The two main principal components (PC1, 77.50%, and PC2, 17.60%) explained 100% of the total variance, demonstrating the distinct separation of the extracts and metabolites and pointing to a high level of similarity between the A and AP extracts (Figure 7).

### 3.5. Greenhouse experiment

#### 3.5.1. Determination of the microbial densities in the soil

**Figure 8.**
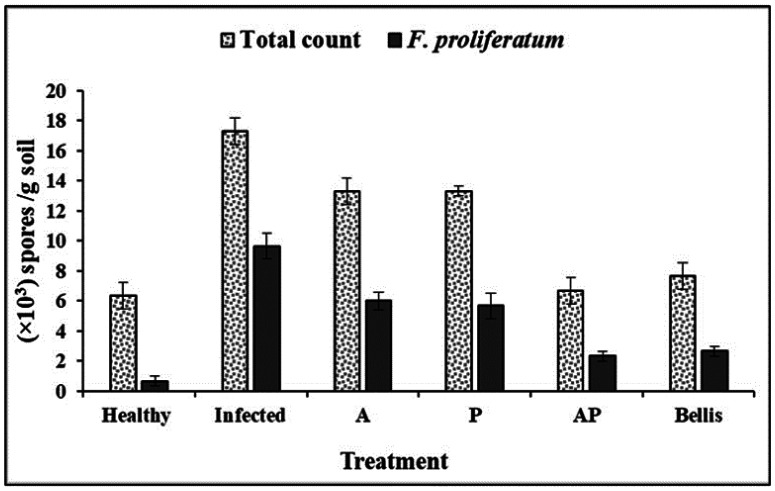
Effect of fungal extracts on the existence of total count of the fungi and *F. proliferatum* in the rhizosphere of onion plants.

The treatment with the AP co-culture extract was superior in reducing the total counts of fungi and *F. proliferatum* as well as the non-infected plants (healthy control), followed by Bellis. Furthermore, a significant decrease in the total count of fungi, the count of *F. proliferatum*, and the percentages of the infected plants was observed with the treatment by AP co-culture extract and Bellis compared to the infected control (6.67 × 10^3^ and 7.67 × 10^3^ total count of total fungi, respectively, while the total count of *F. proliferatum* was 2.33 × 10^3^ and 2.67 × 10^3^, respectively). On the other hand, this decrease gradually increased with other treatments; P and A extracts reported 13.33 and 13.33 × 10^3^ spores/g soil, respectively, for the total count of total fungi, and 5.67 and 6.0 × 10^3^ spores/g soil, respectively, for the total count of *F. proliferatum*. In addition, infected plant percentages were absent in the treatment with AP co-culture extract, and all plants survived (Table S7, Figure 8).

#### 3.5.2. Disease incidence and damage rate

**Figure 9.**
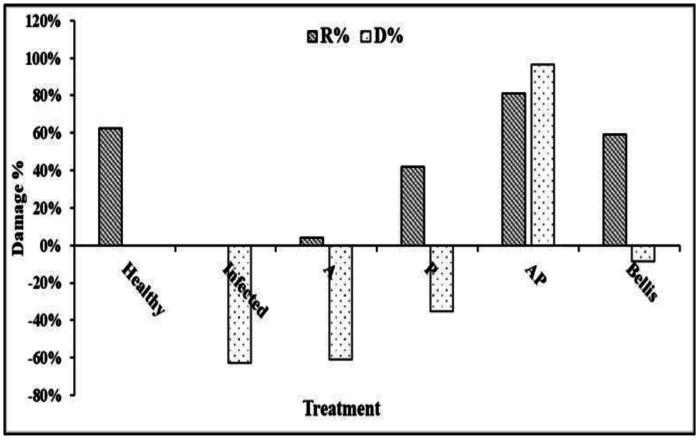
Damage reduction and development rate of the dry shoot and root weights. Development rate (D%) and damage rate (R%).

**Figure 10.**
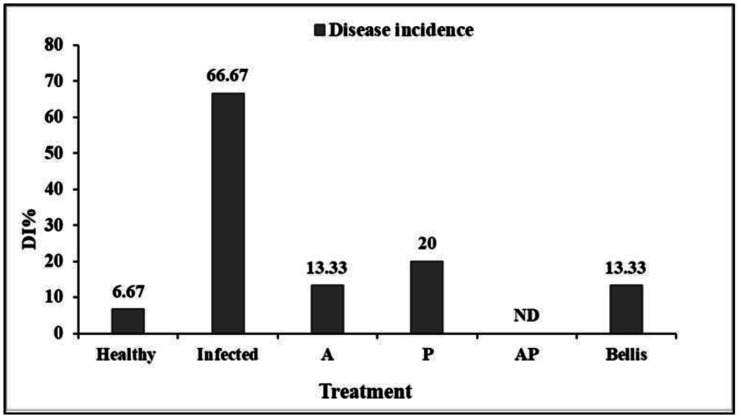
Effect of fungal extracts of monocultures and co-cultures on onion basal rot disease, caused by *F. proliferatum* under field conditions, on disease incidence (%).

The efficacy of onion plants treated individually with different extracts of AP co-culture and monocultures of A and P against onion basal rot disease incidence was evaluated (Table S8). Extract of AP co-culture significantly decreased the disease index (DI) and demonstrated a mean infection frequency of 0.0% compared to 13.30% and 6.67% in Bellis treatment and healthy control, respectively. The damage reduction rate of dry shoot and root weights of onion inoculated by *F. proliferatum* was maximum for AP extract (81% total dry weight) (Figure 9). In contrast, the Bellis treatment reported a damage reduction rate of 59% for dry shoots and roots. The extracts' effects were less noticeable, with values of 4% total dry weight for A culture extract. For onion plants, AP co-culture exhibited the highest development rate (D%), reaching 97% of the total shoot and root dry weights. However, the best behavior of onion plants was observed when they were treated with AP extract compared to healthy control (Figure 10).

#### 3.5.3. Growth parameters

**Figure 11.**
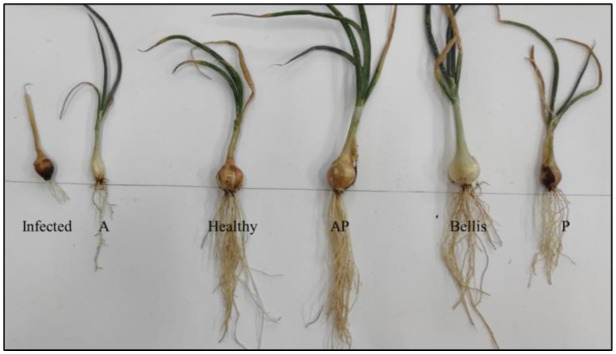
Effect of fungal extracts on the growth parameters of onion plants under greenhouse conditions.

**Figure 12.**
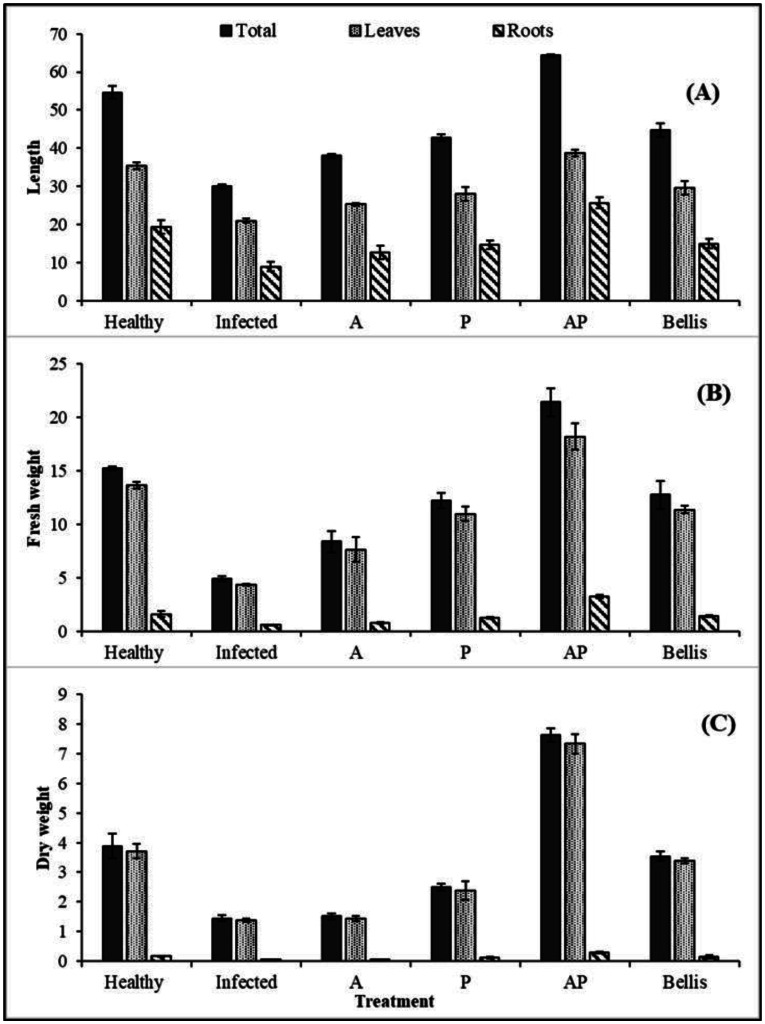
Effect of fungal extracts on the growth parameters of onion plants; (A) height, (B) fresh weight, and (C) dry weight under field conditions.

Growth parameters included total plant height (TH), leaf height (LH), root height (RH), total plant fresh weight (TFW), leaf fresh weight (LFW), root fresh weight (RFW), total plant dry weight (TDW), leaf dry weight (LDW), and root dry weight (RDW) (Table S9, Figures 11 and 12). Infected control plants showed 100% disease incidence, where the bulb was soft, irregular in shape, and discolored at the basal plate with a reduction of 50% in height and 60% in weight compared to the healthy control.

In a greenhouse environment, the effectiveness of fungal mono- and co-culture extracts on growth parameters was investigated. AP co-culture extract treatment showed a significant increase in all parameters for the whole plant, root, and shoot, namely TH (64.33 cm), LH (38.67 cm), RH (25.67 cm), TFW (21.43 g), LFW (18.22 g), RFW (3.21 g), TDW (7.62 g), LDW (7.33 g), and RDW (0.29 g). Low effects on plant parameters were found in treatments with monoculture A and P extracts compared with the healthy control. The healthy control recorded the following parameters: TH (54.67 cm), LH (35.33 cm), RH (19.33 cm), TFW (15.21 g), RFW (1.57 g), LFW (13.64 g), TDW (3.88 g), LDW (3.71 g), and RDW (0.17 g). The infected control recorded the following: TH (30 cm), LH (21 cm), RH (9 cm), TFW (4.89 g), LFW (4.32 g), RFW (0.57 g), TDW (1.45 g), LDW (1.45 g), and RDW (0.06 g).

#### 3.5.4. Photosynthetic pigments content

**Figure 13.**
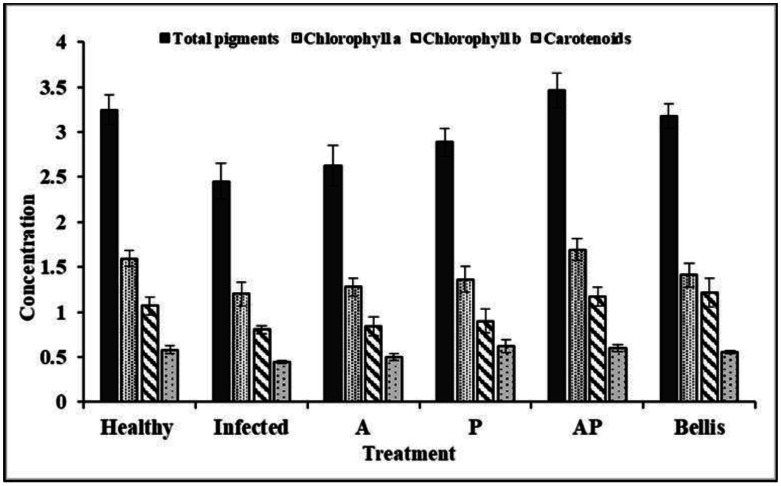
Impact of monoculture and co-culture extracts on photosynthetic pigments.

Table (S10) summarizes the content of various pigments in response to different treatments. The AP co-culture extract treatment led to the highest total pigment content (3.46 mg/g), followed by the Bellis treatment (3.18 mg/g). The infected control displayed the lowest total pigment content of 2.45 mg/g. The AP co-culture treatment had the highest chlorophyll a content (1.69 mg/g), followed by the healthy control (1.59 mg/g). The infected control exhibited the lowest chlorophyll a content (1.20 mg/g). The treatment with Bellis led to the higher chlorophyll b content (1.21 mg/g), followed by the AP co-culture treatment (1.17 mg/g), and the healthy control (1.07 mg/g), while the infected control displayed the lowest chlorophyll b content (0.80 mg/g). Treatment with P culture extract showed the highest carotenoid content (0.62 mg/g), followed by AP co-culture treatment (0.60 mg/g), while Bellis displayed a moderate level (0.55 mg/g). The infected control had the lowest carotenoid content (0.45 mg/g) (Figure 13).

#### 3.5.5. Biochemical compounds

A significant finding was that the AP co-culture demonstrated the highest efficacy in terms of increasing the total soluble carbohydrates of onion leaves (52.10 mg/g dry weight). Furthermore, the healthy control (42.91 mg/g) also exhibited significant enhancements compared to the infected control (31.84 mg/g). The protein content of onion leaves was dramatically raised by the treatment with AP extract (131.44 mg/g), while the lowest protein content was detected in the infected control treatment (75.00 mg/g).

**Figure 14.**
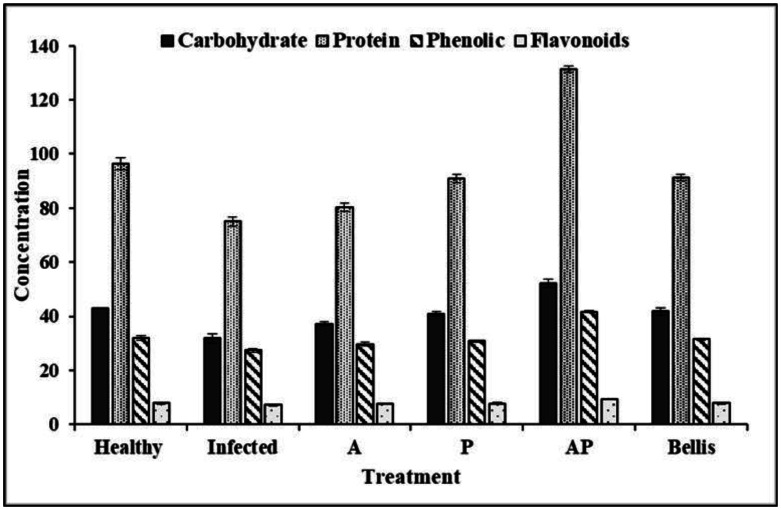
Influence of bioagents on total carbohydrate, protein, phenols, and flavonoids in onion plants.

The phenolic content of leaves treated with co-culture extracts was higher than with mono-culture extract treatments. The maximum increase was detected with the AP co-culture treatment (41.66 mg/g), followed by the chemical fungicide and healthy control (31.45 and 31.93 mg/g, respectively) without significant differences. The lowest phenolic content was detected in the infected control treatment (27 mg/g). Our findings exhibit significant variation of total flavonoids among the various treatments. A maximum yield of total flavonoids was detected with the AP extract (9.43 mg/g), followed by the healthy control (7.85 mg/g). The lowest flavonoid content was detected in the infected control (7.15 mg/g) (Table S11, Figure 14).

**Figure 15.**
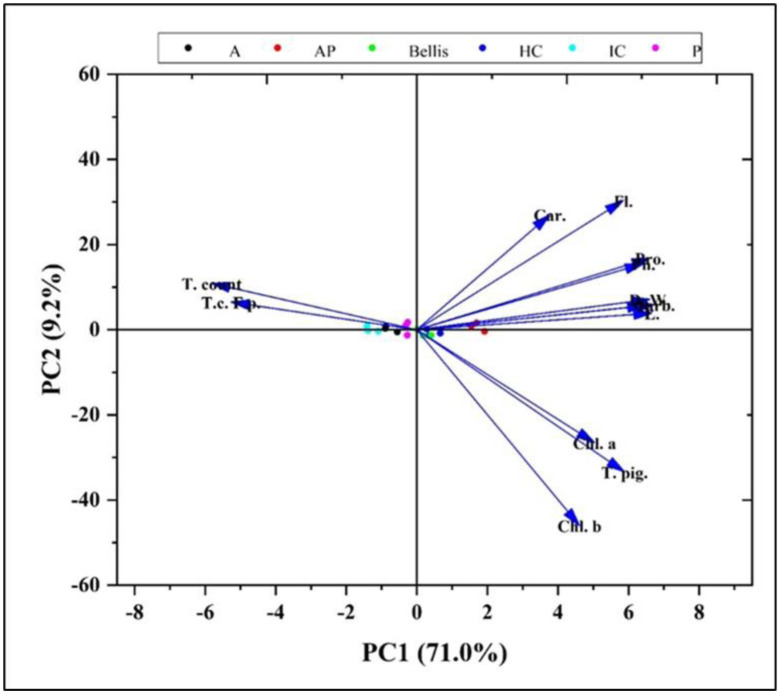
Principal component analysis (PCA) score plot of the data set obtained from various treatments on microbial densities, dry weight, photosynthetic pigments, and phytochemicals in onion.

The principal component analysis revealed two components with eigenvalues greater than one, with group factors relating to microbial densities, dry weight, photosynthetic pigments, and phytochemicals (Table S12). The first factor accounted for 70.99% and the second factor was 9.21%. Factor 1 showed a high loading of factors linked to microbial densities, dry weight, and photosynthetic pigments. Carotenoids, carbohydrates, proteins, phenolics, flavonoids, total dry weight, leaf dry weight, and leaf and root dry weight had strong positive loadings, whereas fungal total count and *F. proliferatum* total count had a negative relationship. Factor 2 components were mostly connected to pigment contents. Components with high factor 2 loadings included Chl. a, Chl. b, and total pigments. The results of the PCA analysis showed that PC1 is a highly significant factor, accounting for a significant portion of the variance in the indices of microbial densities, dry weight, and photosynthetic pigments, influenced by monoculture and co-culture extracts. As shown by the clustering in Figure 15, the infected control (IC) and P extract treatments were strongly correlated with microbial densities indices, with fungal total count and *F. proliferatum* total count having negative values of PC1; these clusters were explained primarily by the high negative values of PC1. On the other hand, the AP co-culture, the Bellis treatment, and the healthy control were substantially linked with growth indicators, photosynthetic pigment contents, and biochemical compounds; these clusters were mostly explained by the high positive values of PC1 and PC2.

## 4. Discussion

The co-cultivation of two or more microorganisms has been shown to be an effective way to enhance the accumulation of known compounds and to induce new secondary metabolites [14]. Rhizosphere and endophytic fungi can alleviate biotic stress by either direct or indirect antagonistic methods [53]. The former relates to the production of certain metabolites that reduce the pathogen population around host plants, whereas the indirect mechanism helps improve crop resistance against phytopathogens [54].

Our study aimed to evaluate the potential of *Aspergillus*-*Penicillium* (AP) co-culture and axenic culture (A and P) extracts to control, in the laboratory and in a greenhouse, onion infection by *F. proliferatum* causing FBR. Molecular identification using ITS genetic sequences of 18S rDNA and phylogenetic characterization of *A. ochraceus* AUMC15539, *P. chrysogenum* AUMC15504, and *F. proliferatum* AUMC15541 was performed. Internal transcribed spacer (ITS) sequences have been utilized extensively to identify species belonging to *Aspergillus*, *Penicillium*, and *Fusarium* genera [55]. Both terminations of the ITS locus revealed nucleotide sequence distinctions in the diverse alignments. Different ITS alignments of tested fungi revealed nucleotide sequence variations, allowing discrimination between fungal species. Both ends of the ITS region revealed nucleotide sequence differences in the multiple alignments. The 5.8S rDNA nucleotide sequences proved to have ideal homology [56]. Mohamed et al. [57] reported that the ITS loci represent essential effective markers for confirming the identification of fungal strains at the species level. Mazrou et al. [58] showed that molecular approaches such as ITS region sequencing were efficient tools for the identification of *Aspergillus* spp. strains.

Our results demonstrated that the AP co-culture extract exhibited significantly higher antifungal activity against *F. proliferatum* (22.17 mm diameter of IZ) compared to the monoculture extracts. The MIC value of the AP co-culture extract was remarkably lower (0.78 mg/mL) than A and P monoculture extracts (12.5 mg/mL), indicating an enhanced antifungal potency in the co-culture. Several polyketides have been isolated from *A. ochraceus* such as mellein and 4-hydroxymellein [59], which exhibit antifungal, antibacterial, and antitumor activities [60],[61]. *Penicillium chrysogenum* has the ability to produce penicillin and small antifungal proteins, making it beneficial for controlling fungal infections [62]. Karpova et al. [63] demonstrated that growth inhibition in four *Fusarium* species occurred by treatment with *P. chrysogenum* dry mycelium.

Regarding cytotoxicity, the AP co-culture extract exhibited the least toxicity to brine shrimp with an LC_50_ value of 2.77 mg/mL, in contrast to the higher toxicity observed in A and P monocultures and Bellis. These findings suggest that the co-culture extract has a better safety profile, making it a promising candidate for further development. Sasidharan and Elyas [64] considered LC_50_ values for brine shrimp toxicity assay lower than 1 mg/mL as toxic. In this regard, Saber et al. [65] reported 100% mortality in brine shrimp larvae when treated with chloroform extract of *Aspergillus ostianus* section Circumdati at a concentration below 1000 µg/mL, thus supporting our result of safe co-culture extracts.

The AP co-culture extract had the highest total phenolic content (114.71 GAE mg/g) and total flavonoid content (27.82 QE mg/g). In comparison, A and P monoculture extracts had lower total phenolic contents of 39.22 and 32.23 GAE mg/g, respectively, and lower flavonoid contents. These elevated levels of phenolics and flavonoids are likely contributors to the enhanced antifungal and antioxidant activities observed. The co-culture extract also demonstrated superior radical scavenging activity (IC_50_ = 1.31 mg/mL), highlighting its potential as a natural antioxidant. Hajdú et al. [66] considered phenols and terpenoids as primary and secondary antioxidants, due to their responsibility in reducing lipid peroxidation, while Chowdhury et al. [67] suggested that the phenolic contents were the major antioxidant constituents of the endophytes. Moreover, Rongai et al. [68] reported a relationship between antifungal activity and total phenolic content, while El Hadrami et al. [69] reported that the inhibition of fungi was mainly due to flavonoids. In contrast, Stanković et al. [70] found that flavonoids were not correlated with antifungal activity.

HPLC analysis revealed significant chemical changes in the co-culture compared to the monocultures, indicating the production of new metabolites. These changes were evident in the altered peak profiles at various wavelengths, suggesting that co-culturing induces the production of unique secondary metabolites that are not present in monocultures. Notably, profiling of AP co-culture and monoculture extracts using HPLC at 235 nm revealed one new peak area at R*t* (10.24); at 340 nm, two new peak areas at R*t* (1.17 and 1.20) were detected only in the AP co-culture extract. Principal component analysis further confirmed the distinct metabolic profiles of the extracts, with the co-culture extract showing a high degree of separation from the monocultures, indicating substantial biochemical differences. Similarly, the HPLC chromatograms of *Trichoderma* and *Talaromyces* monocultures and their co-culture revealed significant differences in peak intensity and retention time, particularly between 5–7 and 9–13 minutes. Notably, a unique peak at 5.9 minutes was observed exclusively in the co-culture chromatogram [71]. The fungus–fungus co-culture strategy can not only stimulate the accumulation of diverse molecules from microorganisms but also significantly increase or decrease the yields of some original secondary metabolites compared to monoculture [72]. Co-cultivation of *Aspergillus* species, *A. fumigatus*, and *A. niger*, led to the regulation of amino acids [73] and increased production of alkaloids [74], meroterpenes [75], polyketides [76], statins, and peptides [77]. These molecules display the potential of the *Aspergillus* genome in the production of highly biologically active metabolites with antimicrobial [76] and cytotoxic activities [78]. Co-culture of *Penicillium pinophilum* and *Trichoderma harzianum* led to the production of compounds by *P. pinophilum* that are not produced in monoculture, such as secopenicillide C, penicillide, and stromemycin [79].

The greenhouse study demonstrated that the AP co-culture extract significantly reduced the incidence of onion basal rot disease and improved plant growth parameters. The AP co-culture extract not only reduced the total count of fungi and *F. proliferatum* in the soil but also decreased the percentage of infected plants to 0%, compared to 13.30% in the Bellis treatment and 6.67% in healthy control. This indicates a strong protective effect of the AP co-culture extract against *F. proliferatum*. In terms of growth parameters, the AP co-culture–treated plants exhibited significant improvements in total growth parameters. These values were notably higher than those recorded for the healthy control and monocultures, emphasizing the co-culture extract's efficacy in promoting plant growth and health.

The AP co-culture treatment resulted in enhancements in photosynthetic pigments that likely contributed to the improved growth and health of the treated plants. A significant finding was that the AP co-culture demonstrated the highest efficacy in terms of increasing the total soluble carbohydrates of onion leaves (52.10 mg/g dry weight) and protein content (131.44 mg/g). The phenolic content of leaves treated with co-culture extracts was also higher, with a maximum increase observed in the AP co-culture treatment (41.66 mg/g) compared with the chemical fungicide and healthy control treatments. Additionally, the AP co-culture treatment yielded the highest total flavonoid content (9.43 mg/g). Endophytic aspergilli are promising reservoirs for bioactive compounds [8]. The utilization of endophytic *Aspergillus* on infected plants led to a noteworthy augmentation in the levels of photosynthetic pigments, total proteins, total carbohydrates, and total phenols when compared with the infected control plants that were not treated [80]. Plant and fungal extracts have been shown to be effective in many plants at inducing systemic resistance, reducing disease incidence, and improving plant growth and production [81].

Moreover, principal component analysis revealed that the AP co-culture, Bellis, and healthy control treatments were substantially linked with growth indicators, photosynthetic pigment contents, and biochemical compounds. The PCA is a statistical method that is included in the more general context of factorial analysis. The PCA allows reducing a set of uncorrelated variables into a smaller number of dimensions [82]. When there are correlations between the descriptive variables of data distribution, the dimensions of the data space exceed the number of characteristic variables necessary to describe these data. The higher the correlations between data descriptive variables, the smaller the number of useful characteristic variables for their representation [83].

## 5. Conclusions

The co-culture approach of *A. ochraceus* and *P. chrysogenum* presents a promising strategy for enhancing antifungal activity against *F. proliferatum* causing onion basal rot and for promoting greenhouse plant growth while maintaining a favorable safety profile over monoculture extracts and chemical fungicide treatments. The significant improvements in antifungal efficacy, phytochemical content, and plant growth parameters demonstrate the potential of co-culture extracts as natural alternatives to conventional fungicides. Further research and development could lead to the commercialization of these extracts, providing sustainable and eco-friendly solutions for managing phytopathogenic fungi in agricultural settings.

## Use of AI tools declaration

The authors declare they have not used Artificial Intelligence (AI) tools in the creation of this article.


