# Supplementary material for: Aspergillus-Penicillium co-culture: An investigation of bioagents for controlling Fusarium proliferatum-induced basal rot in onion
Source: AIMS Microbiol. 2024 Nov 19;10(4):1024–51. doi: 10.3934/microbiol.2024044 (PMC11609423; doi:10.3934/microbiol.2024044)
Supplement: Supplementary file 1 [file microbiol-10-04-044-s001.pdf]

AIMS Microbiology

AIMS Microbiology, 10(4): 1024–1051.
DOI: 10.3934/microbiol.2024044
Received: 26 July 2024
Revised: 03 October 2024
Accepted: 12 November 2024
Published: 19 November 2024

https://www.aimspress.com/journal/microbiology

Research article

Aspergillus-Penicillium co-culture: An investigation of bioagents for controlling *Fusarium proliferatum*-induced basal rot in onion

Mohammed M. M. Abdelrahem[1], Mohamed E. Abouelela[2], Nageh F. Abo-Dahab[1] and Abdallah M. A. Hassane[1,]*

[1] Department of Botany and Microbiology, Faculty of Science, Al-Azhar University, Assiut Branch, Assiut 71524, Egypt
[2] Department of Pharmacognosy, Faculty of Pharmacy (Boys), Al-Azhar University, Cairo 11884, Egypt

* **Correspondence:** Email: abdallahhassane@azhar.edu.eg; Tel: +201122990125.

Supplementary

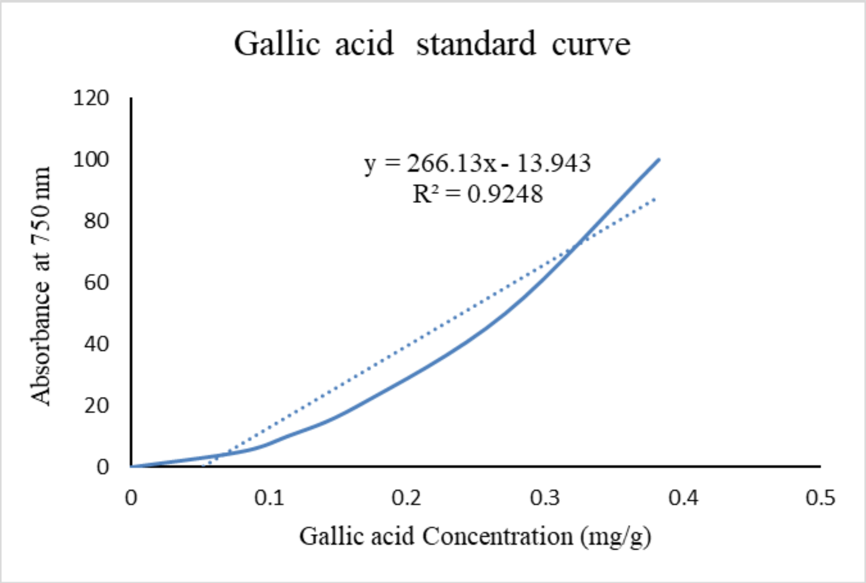

Figure S1. Gallic acid standard cur.

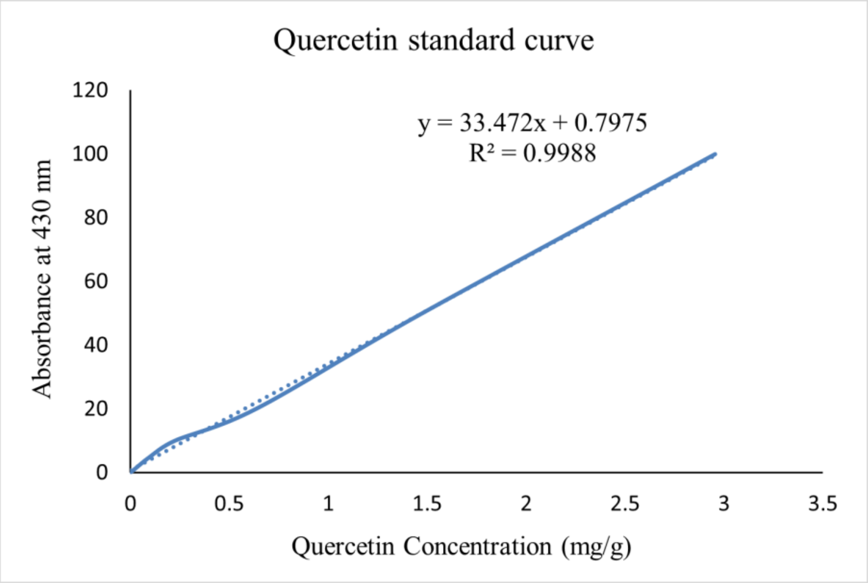

Figure S2. Quercetin standard curve.

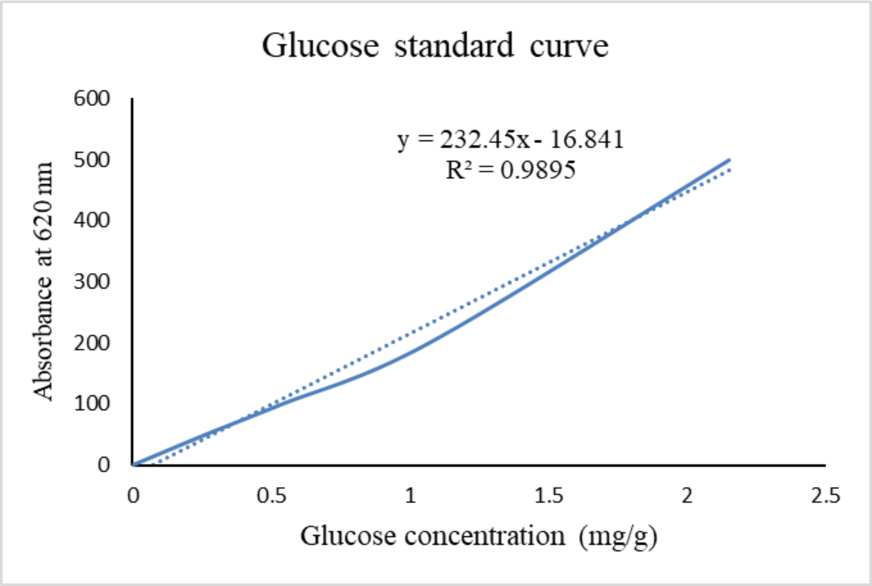

Figure S3. Glucose standard curve.

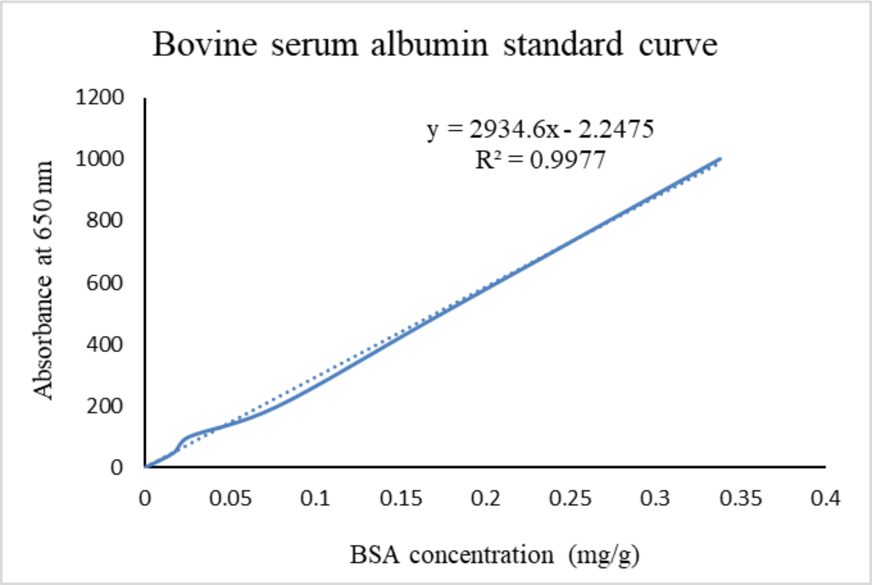

Figure S4. Bovine serum albumin standard curve.

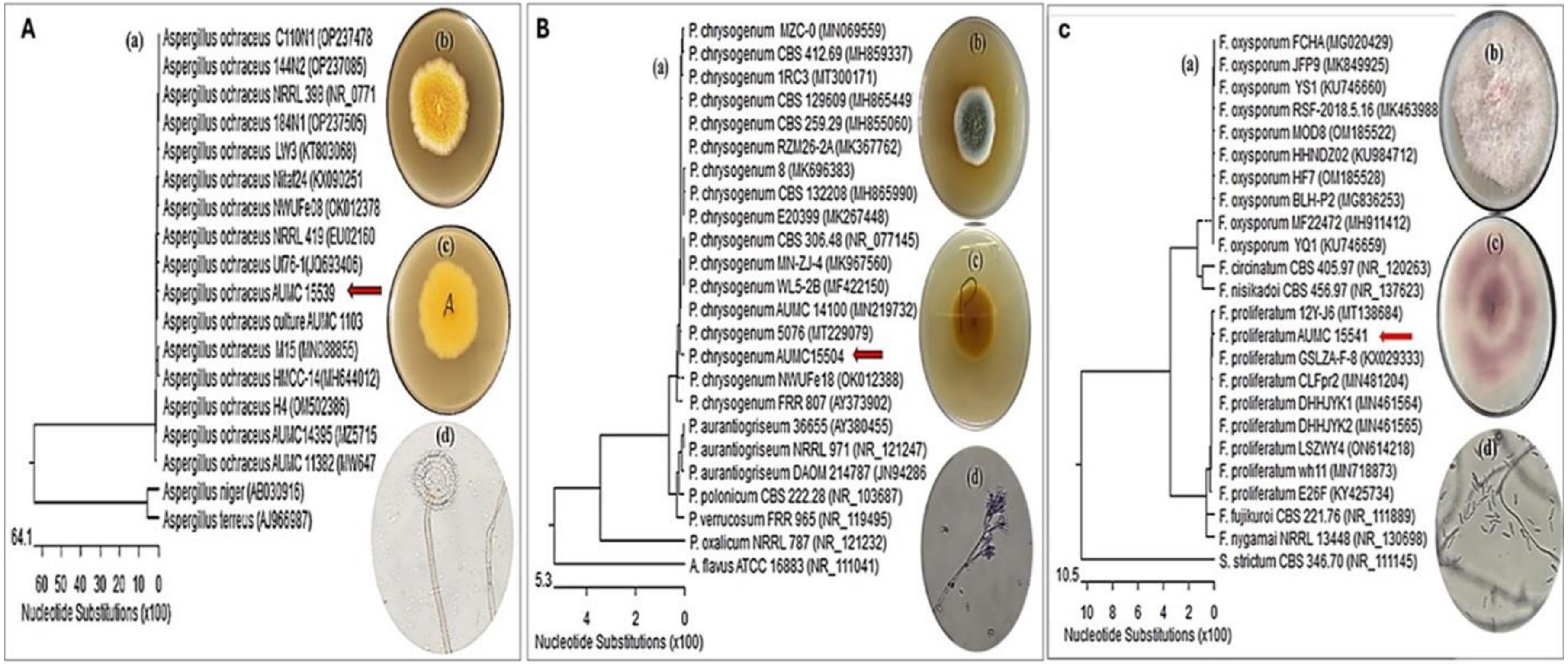

Figure S5. Phylogenetic analysis based on ITS sequences of rDNA of (A) *A. ochraceus* AUMC15539, (B) *P. chrysogenum* AUMC15504, and (C) *F. proliferatum* AUMC15541 aligned with closely similar strains accessed from the GenBank. (a) Phylogenetic tree; (b–d) Culture characteristics, microscope's magnification 400X.

The molecular identity and phylogenetic characterization of *A. ochraceus*, *P. chrysogenum*, and *F. proliferatum* were determined using 18S rDNA gene sequencing (Figure S5A–C). The partial 18S rDNA gene sequence was detected using gel electrophoresis as a clear band at a region of ~541 bps. Sequence homology analysis of *A. ochraceus*, *P. chrysogenum*, and *F. proliferatum* with previously published reference sequences was found using the BLAST tool on the GenBank Database. *Aspergillus ochraceus* aligned with closely similar strains accessed from the GenBank and showed 99.65%–100% identity and 99%–100% coverage with several strains of *A. ochraceus* and showed 100% identity and 100% coverage with the type material NRRL 398 (NR_077150) (Figure S5A). Therefore, the isolated strain wasdesignated as *A. ochraceus* AUMC 15539 with the registered accession number (OR346142). *Penicillium chrysogenum* showed 99.66%–100% identity and 99%–100% coverage with several strains of *P. chrysogenum* including the type material CBS 306.48 (NR_077145) (Figure S5B). Therefore, the isolated strain was designated as *P. chrysogenum* AUMC15504 with the registered accession number (OR343124). *Fusarium proliferatum* aligned with closely similar strains accessed from the GenBank *F. proliferatum* showed 99.63%–99.82% identity and 99%–100% coverage with several strains of *F. proliferatum*. It is worth notable that *F. proliferatum* is considered a synonym for *F. fujikuroi* and showed 99.44% identity and 99% coverage with the type material *F. fujikuroi* CBS 221.76 (NR_111889). Therefore, the isolated strain was designated as *F. proliferatum* AUMC15541 with the registered accession number (OR346141) (Figure S5C).

Table S1. Antifungal activity of ethyl acetate extracts from mono- and co-cultures (100 mg/mL), MIC against phytopathogen *F. proliferatum*, and cytotoxic effect on brine shrimp (LC_{50}).

Extracts	Diameter of IZ (mm) at 100 mg/mL	MIC (mg/mL)	LC_{50} (mg/mL)
A. ochraceus (A)	13.57 ± 0.67^c	12.50	1.83 ± 0.07^b
P. chrysogenum (P)	14.87 ± 0.32^c	12.50	1.42 ± 0.07^d
Co-culture (AP)	22.17 ± 0.26^b	0.78	2.77 ± 0.04^a
Bellis (+ve control)	25.33 ± 0.58^a	1.56	1.68 ± 0.06^c

Data were presented as the mean of three replicates (mean ± SE). Values followed by the different letters are significantly different at $p \leq 0.05$. Inhibition zone (IZ), Minium inhibitory concentration (MIC), and Lethal concentration 50 (LC_{50}).

Table S2. Estimation contents of total phenolic, flavonoid, and IC_{50} values from antioxidant assays of fungal extracts.

Fungal extracts	Total phenolics (GAE mg/g)	Total flavonoids (QE mg/g)	IC_{50} (mg/mL)
A. ochraceus (A)	39.22 ± 0.32^b	18.27 ± 0.12^c	2.92 ± 0.10^c
P. chrysogenum (P)	32.23 ± 0.30^c	19.38 ± 0.15^b	2.84 ± 0.03^c
Co-culture (AP)	114.71 ± 0.57^a	27.82 ± 0.54^a	1.31 ± 0.04^b
BHT*			0.46 ± 0.02^a

Data were presented as the mean of three replicates (mean ± SE). Values followed by the different letters are significantly different at $p \leq 0.05$.

* BHT was employed as antioxidant positive control.

Table S3. HPLC profiles of co-culture (AP) and single cultures of *A. ochraceus* (A) and *P. chrysogenum* (P) at 235 nm.

Peak	R*t*	Area			AP/A%	AP/P%
		A	P	AP		
1	0.94	61.6	0	0	0	0
2	0.97	18.2	0	0	0	0
3	1.43	2454.9	3003.7	2382.8	97	79
4	1.54	73.9	0	0	0	0
5	1.79	2002.1	2107	2099	105	100
6	2.05	0	907.4	800.2	N*	88
7	2.12	1335.8	0	0	0	0
8	2.46	0	684.8	737.1	N*	108
9	2.54	680.3	0	0	0	0
10	2.65	0	116.2	0	0	0
11	2.85	0	20.17	0	0	0
12	3.06	0	16.6	117.3	N*	707
13	3.29	91.9	299.4	22.7	25	8
14	3.60	27.6	0	27.4	99	N**
15	3.72	0	1454.4	351.7	N*	24
16	3.81	351.4	0	0	0	0
17	4.17	34.4	362.9	35.7	104	10
18	4.50	0	118.6	0	0	0
19	4.94	0	1609.9	29.9	N*	2
20	5.11	39.6	484.3	0	0	0
21	5.33	48.9	0	51.2	105	N**
22	5.62	0	98.5	0	0	0
23	5.89	0	86.6	22.2	N*	26
24	6.38	0	96.5	0	0	0
25	6.78	22.1	64.6	25.6	116	40
26	7.11	0	228	0	0	0
27	7.48	42.6	0	44.9	105	N**
28	7.95	0	306	0	0	0
29	8.24	41.2	3721	88.1	214	2
30	9.25	20.1	175	0	0	0
31	10.24	0	0	45	N*	N**
32	10.89	0	36.9	34	N*	92
33	12.22	0	149	123.5	N*	83
34	12.32	0	149	0	0	0
35	18.10	0	69	0	0	0
36	21.62	0	33	0	0	0
37	21.65	0	17	0	0	0

N*: Peak area appeared in AP and not appeared in A.

N**: Peak area appeared in AP and not appeared in P.

Table S4. HPLC profiles of co-culture (AP) and single cultures of *A. ochraceus* (A) and *P. chrysogenum* (P) at 254 nm.

Peak	R*t*	Area			AP/A%	AP/P%
		A	P	AP		
1	1.35	175.1	413.7	193.2	110	47
2	1.41	508.7	904.9	602.9	119	67
3	1.63	22.7	0	0	0	0
4	1.78	349.9	356.1	477.9	137	134
5	2.08	32.2	23.53	0	0	0
6	2.44	158.1	182.6	157.3	99	86
7	3.02	0	24.4	105	N*	430
8	3.26	81.9	62.8	0	0	0
9	3.64	169.2	1685.2	167.6	99	10
10	3.69	72	0	0	0	0
11	4.10	144.37	1109.7	141.4	98	13
12	4.89	0	40.5	0	0	0
13	4.94	79.4	35.9	78.4	99	218
14	5.29	48.4	235.9	47.9	99	20
15	6.73	0	30.7	0	0	0
16	7.09	0	99.9	0	0	0
17	7.92	0	51.6	0	0	0
18	8.21	0	189.4	0	0	0
19	8.64	0	39.7	0	0	0
20	9.22	48.3	125.9	48.1	100	38
21	14.10	98.4	0	97.2	99	N**
22	18.07	0	52.5	0	0	0
23	30.72	63.3	0	135.4	214	N**

N*: Peak area appeared in AP and not appeared in A.

N**: Peak area appeared in AP and not appeared in P.

Table S5. HPLC profiles of co-culture (AP) and single cultures of *A. ochraceus* (A) and *P. chrysogenum* (P) at 280 nm.

Peak	R*t*	Area			AP/A%	AP/P%
		A	P	AP		
1	1.35	0	248	0	0	0
2	1.40	533	653	549	103	84
3	1.63	35	0	35	71	N**
4	1.78	237	242	215	91	89
5	2.04	75	121	66	88	55
6	2.45	113	143	110	97	77
7	3.26	57	504	51	89	10
8	3.49	49	0	57	116	N**
9	3.74	190	946	211	111	22
10	4.09	54	623	79	146	13
11	4.44	0	18	0	0	0
12	4.81	0	24	0	0	0
13	5.06	234	56	236	101	421
14	5.29	114.7	985	111.8	97	11
15	6.37		33	0	0	0
16	6.80	63.9	51	64.7	101	127
17	7.09	0	61	0	0	0
18	8.26	0	41	0	0	0
19	9.25	78.9	286	83.7	106	29
20	14.05	48.4	0	79.4	164	N**
21	30.74	99.7	0	157.7	158	N**
22	30.77	55.7	0	0	0	0

N*: Peak area appeared in AP and not appeared in A.

N**: Peak area appeared in AP and not appeared in P.

Table S6. HPLC profiles of co-culture (AP) and single cultures of *A. ochraceus* (A) and *P. chrysogenum* (P) at 340 nm.

Peak	R*t*	Area			AP/A%	AP/P%
		A	P	AP		
1	1.176	0	0	18	N*	N**
2	1.20	0	0	5	N*	N**
3	1.39	86	63	51	59	81
4	1.47	32	27	25	78	93
5	1.65	143	120	131	92	109
6	1.89	0	25	0	0	0
7	1.95	0	5	0	0	0
8	2.00	0	4	85	N*	2125
9	2.03	0	7	0	0	0
10	6.74	36	58	35	97	60
11	10.88	46	0	46	100	N**

N*: Peak area appeared in AP and not appeared in A.

N**: Peak area appeared in AP and not appeared in P.

Table S7. Effect of fungal extracts on the existence of total count of the fungi and *F. proliferatum* in the rhizosphere of onion plants.

Extracts	Total count of total fungi ($\times 10^3$) spores/g soil	Total count of total *F. proliferatum* ($\times 10^3$) spores/g soil
Healthy control	6.33 ± 0.88^c	0.67 ± 0.33^c
Infected control	17.33 ± 0.88^a	9.67 ± 0.88^a
A. ochraceus (A)	13.33 ± 0.88^b	6.00 ± 0.58^b
P. chrysogenum (P)	13.33 ± 0.33^b	5.67 ± 0.88^b
Co-culture (AP)	6.67 ± 0.88^c	2.33 ± 0.33^{cd}
Bellis (+ve control)	7.67 ± 0.88^c	2.67 ± 0.33^c

Data were presented as the mean of three replicates (Mean ± SE). Values followed by the different letters are significantly different at $p \leq 0.05$.

Table S8. The beneficial effect of fungal mono- and co-culture extracts, on onion plants assay.

Extracts	DW (g)	R%	D%	DI%
Healthy control	3.88 ± 0.41^b	63	0	6.67
Infected control	1.45 ± 0.11^c	0	−63	66.67
A. ochraceus (A)	1.51 ± 0.10^c	4	−61	13.33
P. chrysogenum (P)	2.50 ± 0.11^{bc}	42	−35	20.00
Co-culture (AP)	7.62 ± 0.23^a	81	97	0.00
Bellis (+ve control)	3.54 ± 0.16^b	59	−9	13.33

Data were presented as the mean of three replicates (mean ± SE). Values followed by the different letters are significantly different at $p \leq 0.05$.

Table S9. The effect of fungal extracts on the growth parameters of onion plants under field conditions.

Extracts	Plant height (cm)*			Fresh weight (g)*			Dry weight (g)*		
	Total	Leaves	Roots	Total	Leaves	Roots	Total	Leaves	Roots
Healthy control	54.67 ± 1.76[b]	35.55 ± 0.88[a]	19.33 ± 1.86[b]	15.21 ± 0.17[b]	13.64 ± 0.29[b]	1.57 ± 0.27[b]	3.88 ± 0.41[b]	3.71 ± 0.24[b]	0.17 ± 0.01[b]
Infected control	30.00 ± 0.58[e]	21.00 ± 0.58[d]	9.00 ± 1.15d[d]	4.89 ± 0.25[e]	4.32 ± 0.09[e]	0.57 ± 0.04[c]	1.45 ± 0.11[c]	1.39 ± 0.06[c]	0.06 ± 0.01[d]
A. ochraceus (A)	38.00 ± 0.58[d]	25.33 ± 0.33[c]	12.67 ± 1.86[cd]	8.38 ± 1.01[d]	7.62 ± 1.15[d]	0.76 ± 0.08[c]	1.51 ± 0.10[c]	1.44 ± 0.09[c]	0.07 ± 0.00[cd]
P. chrysogenum (P)	42.67 ± 0.88[c]	28.00 ± 1.73[bc]	14.67 ± 1.20[bc]	12.21 ± 0.73[c]	10.96 ± 0.68[c]	1.25 ± 0.06[b]	2.50 ± 0.11[bc]	2.38 ± 0.31[bc]	0.17 ± 0.02[bcd]
Co-culture (AP)	64.33 ± 0.33[a]	38.33 ± 0.88[a]	25.67 ± 1.45[a]	21.43 ± 1.32[a]	18.22 ± 1.23[a]	3.21 ± 0.15[a]	7.62 ± 0.23[a]	7.33 ± 0.32[a]	0.34 ± 0.03[a]
Bellis (+ve control)	44.67 ± 1.76[c]	29.67 ± 1.76[b]	15.00 ± 1.15[bc]	13.40 ± 1.32[bc]	11.35 ± 0.35[bc]	1.38 ± 0.07[b]	3.54 ± 0.16[b]	3.39 ± 0.10[b]	0.10 ± 0.05[bc]

* Shoot and root height (cm), shoot and root fresh weight (g), shoot of root dry weight (g), Data were presented as the mean of three replicates (mean ± SE). Values followed by the different letters are significantly different at $p \leq 0.05$.

Table S10. Impact of single- and co-culture extracts on photosynthetic pigments.

Treatments	Total pigments	Chlorophyll a	Chlorophyll b	Carotenoids
Healthy control	3.24 ± 0.17^{a}	1.59 ± 0.09^{ab}	1.07 ± 0.10^{ab}	0.58 ± 0.04^{ab}
Infected control	2.45 ± 0.19^{c}	1.20 ± 0.13^{b}	0.80 ± 0.05^{b}	0.45 ± 0.02^{b}
A. ochraceus (A)	2.62 ± 0.23^{bc}	1.28 ± 0.10^{b}	0.84 ± 0.11^{ab}	0.50 ± 0.03^{ab}
P. chrysogenum (P)	2.89 ± 0.15^{abc}	1.36 ± 0.14^{ab}	0.90 ± 0.13^{ab}	0.62 ± 0.07^{a}
Co-culture (AP)	3.46 ± 0.19^{a}	1.69 ± 0.12^{a}	1.17 ± 0.11^{ab}	0.60 ± 0.04^{a}
Bellis (control +ve)	3.18 ± 0.14^{ab}	1.41 ± 0.13^{ab}	1.21 ± 0.16^{a}	0.55 ± 0.02^{ab}

Data were presented as the mean of three replicates (mean ± SE). Values followed by the different letters are significantly different at $p \leq 0.05$.

Table S11. Total carbohydrates, proteins, phenolics, and flavonoids in onion plants as affected by different tested bioagents.

Treatment	Carbohydrates	Proteins	Phenolics	Flavonoids
Healthy control	42.91 ± 0.23^{b}	96.42 ± 2.31^{b}	31.93 ± 0.78^{b}	7.85 ± 0.28^{b}
Infected control	31.84 ± 1.69^{d}	75.00 ± 1.73^{e}	27.34 ± 0.52^{d}	7.15 ± 0.31^{b}
A. ochraceus (A)	37.16 ± 0.73^{c}	80.28 ± 1.53^{d}	29.64 ± 0.73^{c}	7.67 ± 0.22^{b}
P. chrysogenum (P)	40.97 ± 0.92^{b}	90.94 ± 1.58^{c}	30.79 ± 0.48^{bc}	7.75 ± 0.43^{b}
Co-culture (AP)	52.10 ± 1.46^{a}	131.44 ± 1.19^{a}	41.66 ± 0.29^{a}	9.43 ± 0.03^{a}
Bellis (+ve control)	41.75 ± 1.39^{b}	91.33 ± 1.20^{c}	31.45 ± 0.34^{b}	7.81 ± 0.14^{b}

Data were presented as the mean of three replicates (mean ± SE). Values followed by the different letters are significantly different at $p \leq 0.05$.

Table S12. Principal component analysis (PCA) scores.

Properties	Abbreviations	0.20–0.40 m	
		1	2
Total pigments	T. pig.	0.357	0.888
Chlorophyll a	Chl. a	0.324	0.738
Chlorophyll b	Chl. b	0.107	0.905
Carotenoids	Car.	0.651	0.059
Carbohydrates	Carb.	0.792	0.551
Proteins	Pro.	0.874	0.425
Phenolics	Ph.	0.842	0.422
Flavonoids	Fl.	0.909	0.214
Fungal total count	T. count	−0.546	−0.645
F. proliferatum total count	F. pro.	−0.523	−0.549
Total dry weight	T. W.	0.790	0.527
Leaf dry weight	Le.	0.768	0.566
Root dry weight	R.	0.764	0.532
	Total	9.230	1.197
	% of Variance	70.999	9.207
	Cumulative %	70.999	80.205

AIMS Press

© 2024 the Author(s), licensee AIMS Press. This is an open access article distributed under the terms of the Creative Commons Attribution License (https://creativecommons.org/licenses/by/4.0)
